# Evaluating the association between duration of breastfeeding and fine motor development among children aged 20 to 24 months in Butajira, Ethiopia: a case-control study

**DOI:** 10.1186/s12887-023-04391-6

**Published:** 2024-03-27

**Authors:** Rediate Shiferaw, Robel Yirgu, Yalemwork Getnet

**Affiliations:** 1 Department of Reproductive Health and Nutrition, School of Public Health, College of Medicine and Health Sciences, Mizan-Tepi University, Mizan Teferi, Ethiopia; 2 Department of nutrition and dietetics, School of Public Health, Addis Ababa University, Addis Ababa, Ethiopia

**Keywords:** Breastfeeding duration, Developmental delay, Fine motor development

## Abstract

**Background:**

A Suitable environment and proper child nutrition are paramount to a child’s physical and mental development. Different environmental factors contribute to proper child development. Breast milk is an important source of nutrition during the early years of life and contains essential nutrients that are the building blocks for growth and development.

**Objective:**

To assess the association between the duration of breastfeeding and fine motor development among children aged 20 to 24 months living in Butajira, southern Ethiopia.

**Method:**

Community-based case-control study design was employed among mother-child dyads of children aged 20 to 24 months in Butajira Southern Ethiopia. Children were screened for fine motor delay using the Denver II developmental screening and identified as cases and controls. A repeated visit was done to gather the rest of the information and 332 samples, 83 cases, and 249 controls were available and assessed. Epi-data version 4.4.2.1 software was used to prepare a data entry template, which was later exported to and analyzed using STATA version 14 statistical software. Finally, a Multivariable logistic regression model was used to adjust for confounders and estimate the independent effect of breastfeeding duration on fine motor development.

**Result:**

We didn’t find a significant association between the duration of breastfeeding from 21 to 24 months and fine motor delay compared to children who were breastfed less than 18 months[AOR: 0.86, 95% CI: (0.36, 2.05)]. Children who have mothers > 35 years of age were 78% less likely than children who had mothers younger than 25 years, Children who had mothers in secondary school and above were 77% less likely than mothers who didn’t have formal education, Females were 1.86 times more likely than males, and Children who scored 20–29 on the Home score were 51% less likely than Children who scored < 20 to have fine motor delay.

**Conclusion:**

Duration of breastfeeding was not significantly associated with fine motor delay for children aged 20 to 24 months old. The age of the mother, the educational status of the mother, being female, and Home score were identified to have a significant association with fine motor delay. Improving the educational status and empowerment of women is essential. Further work should be done on avoiding gender differences starting from a young age and creating a conducive environment for child development is crucial.

## Background

The first 2 years can predict the quality of life a child can have. Appropriate connections are formed and wired in this window if this stage is passed then it is hard to rewire the brain connections [1]. Child development is a dynamic process that is a result of the interaction between biological and environmental factors [2]. Motor development is also seen as an indicator of global child development [3]. Motor development is the development of the child’s bones, muscles, and ability to move around and manipulate their environment [4]. Motor development is a critical factor in child behavior, being associated with the foundation of cognitive and social-emotional development[3]. Fine motor development is very important for the development of gross motor skills and is connected to how a child performs later on other cognitive tasks, reading and solving mathematical problems [5]. Fine motor skill is the ability to control movement through activities and coordination of the nervous system, fibril, and muscles such as fingers and hands [6]. Fine motor skills are important to do certain activities such as eating and handwriting. The United Nations has set sustainable developmental Goals to improve early child development by 2030. Goal 4 Target 4.2 supplies all children to get access to quality early childhood development so they are ready for primary education [7].

Child development is a dynamic process that is a result of the interaction between biological and environmental factors. Although infant development is influenced by several factors, The centrality of good nutrition cannot be ignored by providing the important building blocks for development [2]. Breastfeeding is the main source of important nutrients for children at this age. Breastfeeding has been identified by the World Health Organization (WHO) as an ideal source that contains important nutrients that can help for the optimal growth and development of children. WHO recommends continuing to breastfeed for up to 2 years with additional complementary foods [8]. Especially fatty acids Docosahexaenoic acid and Arachidonic acid in breast milk are important for brain growth and development and the formation of important synapses or connections in the brain. When a child is adequately nourished with important nutrients in the foundational period it creates a base for lifetime proper brain function [9]. Motor skills are also affected by factors such as caregiving practice, and stimulating environments [1]. Nutritional supplementation and psychosocial stimulation together result in greater improvements in child development than either intervention alone [9]. Determining the solo influence of breastfeeding on child development is difficult because child development is interrelated and associated with different environmental and biological factors. The complexity of child development makes it difficult to evaluate these effects [10]. The effects of environmental factors are pronounced in areas with limited access to the requirements for development [11]. Especially in developing countries, the problem can be worse due to limited resources in the environment that can aggravate the problem [12]. In resource-limited environments, limited resources such as poor stimulating environments and poor nutrition can limit the developmental potential of the children. Therefore, we need to study the effect of multiple environmental factors and nutrition on child development especially in a developing country context. To our best knowledge there are limited studies regarding developmental delays and also the practice of assessing child development in Ethiopia is low. Therefore, knowing the current status and assessing the impact is helpful for early intervention to prevent different adverse outcomes.

## Materials and methods

### Study design, area, and period

Community-based Case-control study was conducted from March to May 2019 among children aged 20 to 24 months living in Butajira Health and demographic surveillance site located in Southern Nations and Nationalities (SNNP), Ethiopia. The area is located 135 km south of Addis Ababa and 50 km to the west of Zeway town in the Rift Valley, 8.2^o^ north latitude and 38.5^o^ east longitude. The climate varies from arid dry lowland areas at altitudes around 1,500 m (tropical climate) to cool mountainous areas up to 3,500 m above mean sea level (temperate climate). The livelihood of the residents is based on mixed farming. Khat (Catha edulis Forsk) and chili-peppers are the main cash crops, while maize and “false banana” or Ensete (Ensete ventricosun) are the main staples [13].

### Source population

All children within the age group of 20 to 24 months living in Butajira HDSS are the Source population.

### Study population

Children within the age group of 20 to 24 months living in Butajira HDSS have been identified as cases and controls based on the Denver developmental screening test.

### Case definition

Cases were children who were identified as being suspect for fine motor delay and controls were children without fine motor delay.

**Case (Suspect**): Two or more cautions (Item on which the age line fails or between the 75th and 90th percentile). This means 75% of the children can pass the test below the child’s age, and /or One or more delays (a child fails to perform an activity that fails completely to the left of the age line) using the Denver developmental screening test.

It is considered that a child fails to perform an item that 90% of children can perform at an earlier age.

#### Control (normal)

No delays and a maximum of one caution using the Denver developmental screening test.

### Study variables

The **outcome variable** was Fine motor delay. The **exposure variables** were Nutritional factors (Breastfeeding duration, Dietary habits of the infant), Child characteristics (sex of the child, birth order), Socio-demographic variables (age of mother, occupation of mother, education status of mother, socioeconomic status), and Caregiving practice: (Home environment.)

### Sample size calculation

The required sample size was calculated using EPI INFO 7 software using an unmatched case-control study using Proportion of Controls among those who breastfeed < 6 months P = 89.97% and Proportion of Cases among those who breastfeed < 6 months P = 76.4% and OR = 0.36 A study done in Taiwan [14].

At precision level of 5%, 95% confidence interval, and 80% power and using r = 3(ratio of cases to controls) and 10% for non-response compensation the sample size becomes 360 with 271 controls and 90 cases.

### Sampling method

**A survey (screening)** was conducted from March to May 2019 in Butajira HDSS by obtaining a sampling frame from the Butajira HDSS. Participants were all children from 20 to 24 months living in the Butajira HDSS. The total population of children in Butajira HDSS from 20 to 24 months was 453.

After going into each Keble and household 376 children that were available were assessed using the Denver developmental screening test and identified as cases and controls. We found 85 cases and 291 controls. Then after identifying the households with cases and controls a repeated visit on the same household and on the same child was done to gather the rest of the information. After visiting the households 332 samples 332 samples 83 cases and 249 controls were available and were assessed using interviewer-administered questioners.

### Operational definition

#### Caregiver (caretaker)

The people who look after infants and young children [15].

#### Breastfeeding less than 18 months

mothers while in the data collection period report that they have breastfed their babies less than 18 months.

#### Continue to breastfeed 18 to 20 months

mothers while in the data collection period report that they have breastfed their babies from 18 to 20 months and stopped.

#### Continue to breastfeed 21 to 24 months

mothers while in the data collection period report that they have breastfed their babies from 21 to 24 months.

### Fine motor development

The fine motor section of Denver II contains 33 items. Each test item on Denver II is presented on a chart by a horizontal bar partitioned into 25, 50, 75 and 90 percentile ages of passing the items. After calculating the exact age draw the age line after drawing the age line the child was asked to perform an activity to the left of the age line, this was done until the child was able to pass three or more consecutive items. Also, the child was tested for items above the age line until the child failed three or more consecutive items.

A child can pass-fail or refuse an item on which the age line fails.

By then identifying the child’s outcome using all the scores that the child has and finding the results will be carried out.

The scoring has 4 items.

“P” for pass – the child successfully performs the item or the caregiver reports (when appropriate) that the child does the item.

“F” for fail- the child does not successfully perform the item, or the caregiver reports (when appropriate) that the child does not do the item.

“N.O” for no opportunity- the child has not had the opportunity to perform the item, due to restrictions from the caregiver or other reasons. This score may only be used on “report” items.

“R” for refusal- the child refuses to attempt the item. Refusal can be minimized by telling the child what to do rather than asking. If given instruction in proper administration, the caregiver may administer the item. Report items cannot be scored as refusals.

#### Normal

no delays and a maximum of one caution.

**Caution** items are interpreted when a child fails or refuses an item on which the age line fails or between the 75th and 90th percentile. This means 75% of the children can pass the test below the child’s age.

**Delays** are considered when a child fails to perform an activity that fails completely to the left of the age line. (Not on the item that the age line passes) It is considered that a child fails to perform an item that 90% of children can perform at an earlier age.

This means 75% of the children can pass the test below the child’s age. When a child passes, fails, or refuses an item that is between the 25th and 75th percentile it is considered normal.

#### Delays suspect

Two or more cautions and /or one or more delays.

Caution items are interpreted when a child fails or refuses an item on which the age line fails or between the 75th and 90th percentile. This means 75% of the children can pass the test below the child’s age [16].

#### Adequate dietary diversity

Children who receive foods from 4 or more food groups using 24-hour recall [8].

#### Inadequate dietary diversity

Children who received foods less than four groups using 24-hour recall [8].

**Household wealth index**—is households’ living status and was constructed by using household asset data on housing conditions like the type of floor, the material of the wall, the material of roof; ownership of assets like radio, TV, telephone, vehicle; the presence of functional latrine, source of drinking water, ownership of domestic animals, ownership of farmland and amount of grain harvested in the last production year among others. After running principal components analysis (PCA) in STATA, the households’ wealth index was grouped into quintiles (lowest quintile, second quintile, middle quintile, fourth quintile, and highest quintile).

### Data collection instrument and procedure

Development was assessed by the Denver developmental screening test which is designed to test the development of the child. The data collection started by Screening for a suspect for fine motor development. The fine motor was assessed using the Denver II developmental screening test. The tool contains different materials that help to examine the development of the child and a test form that contains all the developmental domains in sections. The Denver II tool was adapted in Jimma into a developing country context and was validated in Butajira Ethiopia [17, 18]. The Denver II was assessed by a BSC nurse trained and certified for assessing children using the Denver developmental screening test.

The test was done in a natural and comfortable environment where the child could play with minimal disturbance in the presence of the caretaker. The test was started by informing the mother that the child is not expected to pass all the items.

The test contains a total of 125 items in four developmental domains: personal-social, fine motor, language, and gross motor. The fine motor section of Denver II contains 33 items. Each test item on Denver II is presented on a chart by a horizontal bar partitioned into 25, 50, 75 and 90 percentile ages of passing the items.

Draw the exact age without rounding off days, weeks, or months. Age scales are placed at the top and bottom of the page. Spaces between the age marks represent 1 month until 24 months. After carefully identifying the child’s age draw the age line using the age scales draw an age line from the top to the bottom of the form. After drawing the age line the child was asked to perform an activity to the left of the age line, this was done until the child was able to pass three or more consecutive items. Also, the child was tested for items above the age line until the child failed three or more consecutive items.

For each item, there are 25th, 50th, 75th and 90th percentile.

The age line, pass through the following tasks.

16. Dump coffee bean demonstrated.

Show the child 2 or 3 times how to dump the coffee bean out of the bottle. Then ask the child to get it out. (Do not use the word “dump.”)

Pass if the child dumps the coffee bean out of the bottle or rakes the coffee bean close to the opening and then dumps it out. Do not pass if the child removes the coffee bean with a finger.

17. Tower of cubes – 2, 4, 6, 8.

With the child sitting high enough at the table so that elbows are level with table top and hands are on the table, place the blocks on the table in front of the child. Encourage the child to stack them by demonstration and words. It may be helpful to hand the blocks to the child, one at a time. Three trials may be given.

Pass *Tower of 2 cubes* if the child puts one block on top of another so that it does not fall when he/she removes his/her hand.

Pass *Tower of 4, 6, 8 cubes*, depending upon the greatest number of blocks the child stacks in three trials.

A pass of *4, 6, or 8 cubes* also passes the lower tower items (for example, passing *Tower of 6 cubes* also passes *Tower of 2 and 4 cubes*).

By then identifying the child’s outcome using all the scores that the child has and finding the results will be carried out.

The scoring has 4 items.

“P” for pass – the child successfully performs the item or the caregiver reports (when appropriate) that the child does the item.

“F” for fail- the child does not successfully perform the item, or the caregiver reports (when appropriate) that the child does not do the item.

“N.O” for no opportunity- the child has not had the opportunity to perform the item, due to restrictions from the caregiver or other reasons. This score may only be used on “report” items. “R” for refusal- the child refuses to attempt the item. Refusal can be minimized by telling the child what to do rather than asking. If given instruction in proper administration, the caregiver may administer the item. Report items cannot be scored as refusals.

By then identifying the child’s outcome using all the scores that the child has and finding the results were carried out.

#### Normal

no delays (the child successfully performs the action) and a maximum of one caution (between the 75th or 90th percentile).

#### Suspect

two or more cautions and/or one or more delays (the child fails to perform an activity that fails completely to the left of the age line.)

#### Untestable

refusal scores on one or more items completely to the left of the age line or on more than one item intersected by the age line in the 75-90% area.

Praise the child even for items that are failed. This will build the confidence of the child to attempt more difficult items.

Data on socio-demographic, breastfeeding, and nutritional histories were collected using interviewer-administered questions.

Total Breastfeeding duration was assessed from a study that assessed breastfeeding duration since birth [19]. It was taken by asking the mother to recall the total duration she breastfed her child. Whether she is still breastfeeding or to recall the time she stopped breastfeeding her child.

Complimentary food was assessed using WHO dietary diversity [8]. Dietary diversity was collected using dietary diversity scores adapted from the WHO standardized questionnaire for infant and young child feeding (IYCF). Mothers or caregivers were asked to recall all the food items that the child consumed during the past 24 h [8]. The home environment was assessed using the Home inventory used in different studies [20]. The Home score was assessed by interview-administered questionnaires. It was done by giving the mother a picture book and the mother will show the picture book to the child. Observation will be made on the interaction and the response the mother has towards her child. The interview was conducted in a free and friendly environment. The observation was made on the maternal and child interaction and maternal responses towards the child while asking other questions from the Home inventory.

The training was given to data collectors and supervisors regarding the objective and method of data collection and discussions were made for unclear questions in the questionnaire.

### Data processing and analysis

Data were checked manually for completeness and entered into Epi-data version 4.2.2.1 statistical software and exported into STATA version 14 for data cleaning and analysis. Frequencies and summary statistics (median, interquartile range, percentage, and range) were used to describe the study population in relation to relevant variables.

Nutrition-related variables such as duration of breastfeeding were categorized based on the duration of breastfeeding in months and were grouped as breastfed less than 18 months, 18 to 20, and 21 to 24 months. Dietary diversity was also assessed using a Minimum dietary diversity score comparing children who had consumed four or more food groups and children who scored less than four groups using 24-hour recall. Socioeconomic status was analyzed based on the wealth index by using Principal component analysis (PCA). Childcare practices, maternal-child interaction were checked using the Home score.

Binary logistic regression was used to check for the association between the dependent, fine motor delay, and independent variables. Variables with P- value < 0.2 and which had clinical importance or subject matter were included in the multiple logistic regression. In the multiple logistic regressions, Variables with 95% CI of AOR which did not include 1 were considered to have significant association with the outcome variables. The goodness of fit test indicated (P = 0.0518) that the model was good enough to fit the data well.

### Ethical consideration

Before data collection ethical clearance was obtained from Addis Ababa University School of Public Health Institutional Review Board (AAU-IRB). Written Informed consent was obtained from parents (legal guardians) before participating in the study. All study participants were informed about the purpose of the study, their right to deny participation, anonymity, and confidentiality of the information. All methods were carried out in accordance with relevant guidelines and regulations. The Denver II developmental screening test used in this study to measure the developmental milestone was assessed by a well-trained and certified data collector to ensure the safety of the children. It was conducted in a free and friendly environment. It was explained to the parents that the scale determines the child’s current developmental status and that it’s not an IQ test and the child is not expected to pass all the tests administered. The beneficence of the participants was assured by providing education to the participants about the benefits of breastfeeding, growth, and development. For Children identified with developmental delay, further education was given on methods of improving the motor skills of the Children. The confidentiality of the information of the participants was not disclosed.

## Result

### Socio-demographic characteristics of the study participants

Mothers in the age group from 25 to 29 years were 35(42.17%) in the cases while 101(40.56%) were in the controls. The median age of the mothers was 28, IQR (25 33%). About 48(57.83%) of mothers in the cases and 93(37.65%) of mothers in the controls didn’t have any formal education. About 58(69.88%) of the cases and 185(74.60%) of mothers from the controls were Housewives. About 39(46.99%) fathers in the cases and 67(26.91%) in the controls didn’t have any formal education. About 23(27.71%) of the cases and 52(20.88%) in the controls were from the lowest quintile. About 69(83.13%) of the cases and 179(71.89%) of the controls were Rural residents (Table 1).

**Table 1:** Socio-demographic characteristics of the participants in Butajira, Ethiopia, 2019

Characteristics	Fine motor delay	
Socio-demographic characteristics	**Cases (n, %)** **n = 83**	**Controls (n, %)** **n = 249**
Maternal age at birth		
18–24	21(25.30)	61(24.50)
25–29	35(42.17)	101(40.56)
30–34	22(26.51)	64(25.70)
35 and above	5(6.02)	23(9.24)
Mothers education		
No formal education	48(57.83)	93(37.65)
Primary school(1–9)	29(34.94)	108(43.72)
Secondary school(9–12) and above	6(7.23)	46(18.62)
Maternal occupation		
Housewife	58(69.88)	185(74.60)
Merchant	24(28.92)	53(21.37)
Government employee	1(1.20)	10(4.03)
Marital status		
Married	80(96.39)	241(96.79)
Divorced	1 (1.20)	2 (0.80)
Widowed	2(2.41)	6(2.41)
Fathers education		
No formal education	39(46.99)	67(26.91)
Primary school(1–9)	33(39.76)	118(47.39)
Secondary school(9–12) and above	11(13.25)	64(25.70)
Area of residence		
Urban	14(16.87)	70(28.11)
Rural	69(83.13)	179(71.89)
Socioeconomic status (Wealth index in quintile)		
Lower	23(27.71)	52(20.88)
Second	17(20.48)	58(23.29)
Middle	19(22.89)	60(24.10)
Fourth	15(18.07)	38(15.26)
Higher	9(10.84)	41(16.47)

### Child-related characteristics

The study included 168 male and 164 female children from the age group of 20–24 months. About 36(43.37%) males were cases while 132(53.01%) were in the controls (Table 2).

**Table 2:** Child-related characteristics of the participants in Butajira, Ethiopia, 2019

Characteristics	Fine motor delay	
Child characteristics	**Cases (n, %)** **n = 83**	**Controls (n, %)** **n = 249**
Sex of the child		
Male	36(43.37)	132(53.01)
Female	47(56.63)	117(46.99)
First	14(16.87)	47(18.88)
second	8(9.64)	54(21.69)
Third	23(27.71)	43(17.27)
>=4	38(45.78%)	105(42.17)
Relation with the child		
Mother	81(97.59)	243 (97.59)
Caregiver	2(2.41)	6 (2.41)

### Delivery and nutritional characteristics of the study participants

Health facility delivery among the cases was 67(80.72%) and 213(85.54%) among the controls. Breastfeeding at least once was 81(97.59%) among the cases and 248(99.60%) among the controls. About 49(59.04%) mothers in the cases and 139(55.82%) in the controls reported that they are currently breastfeeding.

About 66(79.52%) children in the cases and 177(71.08%) children in the controls continued to be breastfed from 21 to 24 months. There was no significant variation among cases and controls by the duration of breastfeeding 95% CI (p = 0.234) (Table 3).

**Table 3:** Delivery and Nutritional characteristics of the participants in Butajira, Ethiopia, 2019

Characteristics	Fine motor delay		Chi2 (P-value)
Socio-demographic characteristics	**Cases (n, %)** **n = 83**	**Controls (n, %)** **n = 249**	
Place of birth			
Home	16(19.28)	36(14.46)	0.446
Health facility	67(80.72)	213(85.54)	
Mode of delivery			
Spontaneous vaginal delivery	78(93.98)	239(95.98)	0.861
Instrumental delivery	5(6.02)	10(4.02)	
Ever breastfeed			
Yes	81(97.59)	248(99.60)	0.155
No	2(2.41)	1(0.4)	
Fed colostrum			
Yes	78(93.98)	245(98.39)	0.047
No	5(6.02	4(1.61)	
Time of Initiation of Breastfeeding			0.14
Immediately less than an hour	75(90.36)	243(97.98)	
Between 1 and 23 h	5(6.02)	4(1.61)	
More than 24 h	3(3.61)	1(0.40)	
Currently breastfeeding (From 20 to 24 months)			
Yes	49(59.04)	139(55.82)	0.609
No	43(40.96)	110(44.18)	
Frequency of breastfeeding			
Only at night	2(4.08)	2(1.44)	0.372
2 to 3 times during the day	7(14.29)	12(8.63)	
3 to 5 times during the day	22(44.90)	59(42.45)	
6 times and more	18(36.73)	66(47.48)	
Duration of breastfeeding			
Less than 18	11(13.25)	38(15.26)	0.234
18 to 20 month	6(7.23)	34(13.65)	
21 to 24 month	66(79.52)	177(71.08)	

### Dietary practices and nutritional characteristics of the children

About 46(55.42%) of children in the cases and 179(71.89%) in the controls started solid or semi-solid food within 6 to 8 months. There was a difference among cases and controls on children at the time of starting solids and semisolid foods (Table 4).

**Table 4:** Dietary practices of the children aged 20 to 24 months in Butajira, Ethiopia. 2019

Characteristics	Fine motor delay			Chi2(P-value)
	**Cases (n, %)** **n = 83**		**Controls (n, %)** **n = 249**	
Age of starting of solid and semi-solid foods				
> 5 month		10(12.05)	21(8.43)	**0.039**
6–8 month		46(55.42)	179(71.89)	
9-12month		15(18.07)	32(12.85)	
> 12 month		12(14.46)	17(6.83)	
Dietary diversity				
Inadequate diet		76(91.57)	231(92.77)	0.788
Adequate		7(8.43)	18(7.23)	

### Caregiving practice

About 41(49.40%) of children in the cases and 172(69.08%) in the controls had a score between 20 and 29 on the Home score. The Home score had a minimum score of 13 and a maximum score of 32 (Table 5).

**Table 5:** Caregiving practice of the children aged 20 to 24 months in Butajira, Ethiopia. 2019

Characteristics	Fine motor delay		Chi2(P-value)
Home environment	**Cases (n, %)** n = 83	**Cases (n, %)** n = 249	
Less than 20	39(46.99)	72(28.92)	
20 to 29	41(49.40)	172(69.08)	
Greater than 30	3(3. 61)	5(2.01)	

### Association of different characteristics of children with suspect of fine motor delay

In the binary logistic regression variables with p-value < 0.2 or factors that had clinical importance were identified (Table 6).

**Table 6:** Association between child characteristics socio demographic and nutritional characteristics with fine motor delay among children aged 20 to 24 months in Butajira, Ethiopia 2019

Characteristics		Fine motor delay			
		**Case**	**Control**	**COR (95%CI)**	**AOR(95%CI)**
Duration of breastfeeding	Less than 18	11(22.45)	38(77.55)	I	I
	18–20 month	6(15.00)	34(85.00)	0.60(0.20 1.82)	0.45 (0.13 1.56)
	21–24 month	66(27.16)	177(72.84)	1.28(0.62 2.66)	0.86 (0.36 2.05)
Age of the mother at birth	< 25	21(25.30)	61(24.50)	I	I
	25–29	35(42.17)	101(40.56)	1.00 (0.53 1.88)	0.53 (0.22 1.24)
	30–34	22(26.51)	64(25.70)	0.99 (0.49 1.99)	0.39 (0.14 1.11)
	> 35	5(6.02)	23(9.24)	0.63(0.21 1.87)	0.22 (0.05 0.87)*
Educational status of the mother	No formal education	48(57.83)	93(37.65)	I	I
	Primary school(1–9)	29(34.94)	108(43.72)	0.52(0.30 0.89)*	0.34 (0.14 0.81)*
	Secondary and above	6(7.23)	46(18.62)	0.25 (0.10 0.63)*	0. 23 (0.06 0.80)*
Area_of_the_residence	urban	15 (17. 65)	70(82.35)		
	rural	68 (27.53)	179(72.47)	1.92(1.01 3.64)	1.44(0.49 4.21)
Wealth index	Lower	24(31.58)	52(68.42)	I	I
	Second	18(26.47)	50(73.53)	0.78 (0.37 1.60)	0.96 (0.42 2.17)
	Middle	22(28.95)	54(71.05)	0.88(0.44 1.76)	1.11 (0.51 2.42)
	Fourth	11(17.19)	53(82.81)	0.44 (0.20 1.01)	0.78 (0.27 2.27)
	Higher	8(16.67)	40(83.33)	0.43 (0.17 1.06)	1.26(0.30 5.23)
Sex of the child	Male	36(21.43)	132(78.57)	I	I
	Female	47(28.66)	117(71.34)	1.47 (0.89 2.42)	1.86 (1.05 3.28) *
Birth order	First	14(22.95)	47(77.05)	I	I
	Second	8(12.90)	54(87.10)	0.49(0.19 1.28)	0.46 (0.16 1.34)
	Third	23(34.85)	43(65.15)	1.79(0.82 3.92)	1.74 (0.66 4.59)
	>=4	38(26.57)	105(73.43)	1.21(0.60 2.45)	0.81 (0.29 2.26)
Mode of delivery	Spontaneous Vaginal delivery	78(24.61)	239(71.39)	I	I
	Instrumental delivery	5(33.33)	10(66.67)	1.53(0.50 4.61)	1.37 (0.36 5.18)
Starting of solid foods	< 6 month	10(32.26)	21(67.74)	I	I
	6–8 month	46(20.44)	179(79.56)	0.53 (0.23 1.22)	0.56 (0.22 1.39)
	9–11 month	15(31.91)	32(68.09)	0.98 (0.37 2.59)	0.90 (0.30 2.66)
	> 12 month	12(41.38)	17(58.62)	1.48 (0.51 4.25)	1.77 (0.55 5.70)
Dietary diversity	Inadequate	76(24.76)	231(75.24)	I	I.
	Adequate	7(28.00)	18(72.00)	1.18 (0.47 2.93)	1.88 (0.62 5.69)
Home	Less than 20	39(35.14)	72(64.86)	I	I
	20–29	41(19.25)	172(80.75)	**0.44 (0.26 0.73)**	0.49(0.27 0.88)*
	Greater than 30	3(37. 50)	5(62. 50)	1.10(0.25 4.88)	2.5(0.42 15.77)

After adjusting for these variables age of the mother, the educational status of the mother, the sex of the child, and the Home score were identified to have a significant association with fine motor delay.

We didn’t find a significant association between duration of breastfeeding and fine motor delay for children who were breastfed from 18 to 20 months [AOR: 0.45, 95% CI: (0.13, 1.56)] and for children who were breastfed from 21 to 24 months [AOR: 0.86, 95% CI: (0.36, 2.05)] compared to children who were breastfed less than 18 months. Children who have mothers > 35 years of age were 78% less likely to have fine motor delay than mothers who were < 25 years old [AOR: 0.22, 95% CI: (0.05, 0.87)]. Children who had mothers in primary school were 66% less likely [AOR: 0.34, 95% CI: (0.14, 0.81)] and children who had mothers in secondary school and above were 77% less likely [AOR 0. 23, 95% CI: (0.06, 0.80)] to have fine motor delay than mothers who didn’t have any formal education. Females were 1.86 times more likely to have fine motor delay than males [AOR: 1.86, 95% CI: (1.05, 3.28)]. Children who scored 20–29 on the Home score were 51% less likely to have fine motor delay than Children who scored < 20 [AOR: 0.49, 95% CI: (0.27, 0.88)] (Table 6).

## Discussion

Child development is an important aspect of human life. Development can be affected by different factors. Environmental factors and nutritional factors together play a significant role in child development. Nutritional factors have a great role in development but due to the adverse environmental and social factors, the outcome could be influenced by different factors, especially in developing countries [1].

Breastfeeding is known to have a significant effect on child growth and development [11] but in our study, We didn’t find a significant association between the duration of breastfeeding and fine motor delay for children who were breastfed from 18 to 20 and for children who were breastfed from 21 to 24 months compared to children who were breastfed less than 18 months.

Our findings are consistent with some studies that didn’t find a significant association between duration of breastfeeding and fine motor development [21–23]. All the studies acknowledged that breastfeeding is important for development but they suggested that other factors also have a role in influencing fine motor development. Similar to our study, A study in Singapore didn’t find a significant association between breastfeeding and fine motor development at 24 months [21]. Another study done in rural Brazil didn’t find a significant association between breastfeeding and fine motor development at 12 months and suggested home stimulation, maternal education, and income were influencing the outcome [22].

The study in Singapore suggested they have used specific research tools and have controlled for a large number of potential confounders and they didn’t find any relationship between breastfeeding on fine motor development [21]. The study in Brazil investigates the association between breastfeeding and mental and motor development, controlling for comprehensive measures of the child’s socioeconomic maternal, and environmental background, and nutritional status. They didn’t find a significant association between breastfeeding and motor development. They explained that the reason most studies have found an association between breastfeeding and development is that the studies have been done in relatively affluent populations where, in general, mothers who succeed in breastfeeding have higher socio-economic status, better educated with higher educational attainment. While In their study mothers who were breastfeeding longer had lower socioeconomic status, poorer education, and provided less stimulating home environments. They explained the reason that most studies found the association was due to incomplete adjustment for covariates, differences in methodological robustness, and types of tests used are likely to be contributory, which will result in an apparent breastfeeding benefit. To prevent this bias they controlled for different covariates. They suggested that no subgroup is differentially protected by breastfeeding, but rather that all groups benefit. The benefit of breastfeeding was an important factor that benefited all the comparison groups, while it has a beneficial effect, breastfeeding didn’t have a protective effect on fine motor development. The difference in the outcome was appreciated by other potential determinants. They found home stimulation and family income to be more important factors [22].

This is similar to our study finding that mothers who were breastfeeding longer had lower socio-economic status and poorer education. We also have found other environmental factors to be significantly associated. Similar to these studies environmental factors were playing a significant role in fine motor development.

All the studies acknowledged that breastfeeding is important for development but they suggested that other factors were influencing fine motor delay and we need to take into consideration other factors that could also affect or contribute to child development.

A systematic review also suggested that development is influenced by different environmental and psychological factors. Different factors need to be put into consideration that can affect the developmental potential of the children. Their analysis reveals that there are studies that have shown an apparent decrease in effect after multivariate analysis. Given that tight control of confounders resulted in a greater likelihood of the disappearance of the breastfeeding effect. Studies completed in middle-income and low-income countries were nearly twice as likely to find no association compared with studies set in developed countries. The fact that this relationship is less apparent in developing countries suggests that much of the observed relationship may be due to parental social advantage, confounding the choice to breastfeed [23].

In conclusion, the systematic review suggests that much of the reported effect of breastfeeding on child developmental abilities is due to maternal and socioeconomic effects. They suggested additional, future studies in this field are needed to rigorously control for all important confounders [23]. Development is not the solo effect of breastfeeding alone but a combination of different factors working together.

All these studies have used different developmental screening tools so the comparison should be done cautiously.

Contrary to our study A study in Malawi among children who breastfeed from 9 to 10 months found a small but significant protective effect on fine motor development at 12 to 18 months [24]. Studies in Western countries, a study done in Taiwan and Greece assessed the effect of duration of breastfeeding more than 6 months and fine motor assessed at 18 months. They found that any increase in the duration of breastfeeding was associated in decreasing in the odds of fine delay which persisted after controlling for different factors [25, 26]. The Taiwan study has shown that mothers who breastfeed longer were older, had a university education, and were from a better socioeconomic class and suggested that the positive result could be due to the presence of these factors [25]. These factors were different in our setting, the majority of the mothers in this study who breastfeed for longer durations were less educated. Studies have shown that mothers who are more educated create a more favorable and stimulating environment and when breastfeeding is added to these factors there could be better results that can be helpful for child development [27, 28]. This might be one of the reasons why we couldn’t find a significant association.

We have also found the age of the mother to have a significant association with the development of the child. We have found older mothers had more favorable outcomes than young mothers. Similar findings have suggested that older mothers tend to create a more favorable environment for child development and would also breastfeed for longer durations [29–31].

Also, we have found the education level of the mother to be significantly associated with fine motor delay. Children who had mothers in primary and secondary school were less likely to have fine motor delay than mothers who didn’t have formal education. Studies have shown that a mother’s education is important because as the educational level of the mother increases the level of stimuli the mother gives to her child also increases [27]. In addition to that, as the education level of the parents increases the socioeconomic status also could increase and will create a more favorable environment for the children [32].

Another factor that we found significant was the sex of the child. We have found females have greater odds of being affected by fine motor delay than males. Contrary to our study different studies have suggested females have a better score on fine motor and boys have a higher risk of having developmental delay [33, 34]. While we cannot give a general conclusion other factors in the environment could affect the development of females. A study in India has shown that Girls are breastfed for shorter durations than boys due to the gender preferences of the mothers. Mothers will start early weaning for girls than boys to have another pregnancy and not to delay another pregnancy [35]. The gender preferences of the mother could affect the duration of breastfeeding and the care the child will have [36]. This gender preference could lead to a developmental delay in the female population.

We have found the Home score to have a significant association with fine motor development. Similarly, studies found the Home environment to have a significant association with fine motor development [37, 38]. Motor development can be regulated critically by the home environment and maternal and child interaction [39]. The role of the mother or the caregiver has a protective role even for children growing up in limited environments such as low socioeconomic status, low levels of education, chronic illness, conflict, and mental health problems of caregivers. Mothers’ sensitivity is important because it creates a conducive environment for the development of the child [40]. A study done in Iran did not find a significant positive association between home motor affordances and motor development in their sample. They suggested that this could be due to the tool that they used was not sensitive enough to detect differences [3]. Home environment is a very important factor for childhood development a study has shown in nutrition-related interventions certain amount of stimulation from the environment was necessary and nutritional intervention alone was insufficient to bring brain development [40].

The Strengths of this study are the study was a community-based case-control study which is helpful to asses multiple exposure or risk factors. We have also used new cases that were identified at the time of collection which could prevent misclassification bias. We have used tools that are validated in our setting which can measure the case of interest in a better way.

The following limitation needs to be taken into account when interpreting the results. Most of the mothers in our study group had the practice of long-term breastfeeding durations and conducting the study where different information or different study groups are available would help to further strengthen the study finding. Our study was conducted in a rural setting but including the figure of urban mothers would further enrich the information that can be found. Even though birth weight is an important factor for development we didn’t have information on the birth weight of the children.

## Conclusions

This study still supports that breastfeeding is important for child development. However, in our study, we didn’t find a significant association between the duration of breastfeeding from 18 to 20 months and for duration of breastfeeding from 21 to 24 months compared to children who were breastfed less than 18 months on fine motor development. Children from older mothers were less likely to be affected than young mothers. Children who had mothers in primary and secondary school were less likely to have fine motor delay than mothers who didn’t have formal education. Females have higher odds of being suspect of fine motor delay than males. Children who had better maternal care practices or Home scores were less likely to be affected than Children who had lower maternal care practices or lower Home scores.

Based on our findings we forward the following recommendations: Health care providers should be the first-line source of information to provide appropriate information to the mothers and the community during delivery or during any visit the mother makes to the health facility. They should educate the mothers and the community about the importance of child feeding and childcare and creating a conducive environment for child development. Older mothers tend to create more conducive environments for child development. Delaying early pregnancies is helpful to have physically and psychologically mature mothers. Since mothers are the primary caretakers improving maternal education and empowerment to improve developmental outcomes is helpful for child development. Therefore policymakers should work on improving the educational status and empowerment of women and work on avoiding gender differences starting from a young age. Assessment of Developmental delay in children should also be done routinely by Health care providers to catch delays during the early years and to have early interventions. Further studies should be done in a different setup to appreciate the difference and the effect of other environmental factors. Further follow-up studies should be done to prevent recall bias in a better way. Thus, overall, child development can be influenced by different factors in the environment, and having a holistic approach is mandatory to tackle the problem.

## Data Availability

The datasets used and/or analyzed during the current study are available from the corresponding author upon reasonable request.

## List of abbreviations

IYCF: Infant and Young Child Feeding
ARA: Arachidonic acid
DHA: Docosahexaenoic acid
EDHS: Ethiopian Demographic and Health Survey
HDSS: Health and Demographic Surveillance site
SNNPR: Nationalities and Peoples Regional State
WHO: World Health Organization
AOR: Adjusted Odds Ratio
CI: Confidence Interval
COR: Crude Odds Ratio
